# Laser-Assisted Thermal Imprinting of Microlens Arrays—Effects of Pressing Pressure and Pattern Size

**DOI:** 10.3390/ma12040675

**Published:** 2019-02-25

**Authors:** Keisuke Nagato, Yuki Yajima, Masayuki Nakao

**Affiliations:** Department of Mechanical Engineering, Graduate School of Engineering, The University of Tokyo, Tokyo 113-8656, Japan; yajima@hnl.t.u-tokyo.ac.jp (Y.Y.); nakao@hnl.t.u-tokyo.ac.jp (M.N.)

**Keywords:** laser-assisted thermal imprinting, pressure, pattern size, thermoplastic polymer, microlens array

## Abstract

Polymer films with nano- or microstructured surfaces have been widely applied to optical devices, bioplates, and printed electronics. Laser-assisted thermal imprinting (LATI), in which a laser directly heats the surfaces of a mold and a thermoplastic polymer, is one of the high-throughput methods of replicating nano- or microstructures on polymer films. Only the surfaces of the mold and polymer film are heated and cooled rapidly, therefore it is possible to replicate nano- or microstructures on polymer films more rapidly than by using conventional thermal nanoimprinting. In this study, microlens arrays (MLAs) were replicated on polymethylmethacrylate (PMMA) films using LATI, and the effects of the pressing pressure (10−50 MPa) and the pattern size (33- and 5-μm pitch) of the MLA on the filling ratio were investigated by analyzing a microlens replicated using different laser-irradiation times (0.1−2 ms). The filling ratio increased with increasing pressing pressure and laser-irradiation time in the replication of MLAs with varying sizes, while the flow of the PMMA varied with the pressing pressure and laser-irradiation time. It was found that during filling, the shape of the polymer cross-sectional surface demonstrated a double and single peak in the 33- and 5-μm-pitch patterns, respectively. This was because the depth of the heated area in the 33-μm-pitch pattern was smaller than the pattern size, whereas that of the 5-μm-pitch pattern was comparable to (or larger) than the pattern size.

## 1. Introduction

Nano- or microstructured polymer surfaces perform various functions, such as hydrophilic or hydrophobic interactions with liquids, reduction of friction or sticking to solid surfaces, demonstrating an anchoring effect for adhesives, and optical or photonic behaviors. In terms of their use in optical or photonic capacities, antireflection structures have been fabricated to reduce light reflection and to increase the incident light transmittance in solar cells, flat panel displays and so forth [1]. Microlens arrays (MLAs) are fabricated on OLED devices, or OLEDs are fabricated on polymer films with photonic crystal surfaces [2,3,4,5,6] to improve the light extraction efficiency of organic light-emitting diodes (OLEDs). Furthermore, various nanostructures have been fabricated on polymer films for organic photovoltaic cells [7] and nanopillars have been fabricated for plasmonic biosensing [8]. As described above, nano- or microstructures are expected to become more commonly applied to various optical and photonic devices.

For these applications, it is necessary to fabricate nano- or microstructures on polymer films with a high throughput and low cost. Nanoimprint lithography (NIL), which was developed by Chou et al., is one of the highest-throughput and most inexpensive methods of fabricating nano- or microstructures [9]. To obtain nano- or microstructured surfaces on polymer films, three main kinds of nanoimprint method have been proposed and developed: thermal nanoimprinting [9], ultraviolet (UV) nanoimprinting [10], and soft lithography [11]. UV lithography involves the use of a UV-curable polymer which is coated and molded and then cured using UV light. Soft lithography involves the use of thermocurable polymer coatings, which are cured post heating. These lithography techniques possess advantages associated with the use of soft molds and the use of low applied pressures because of the low viscosity of polymers. Flexible substrates can thus be coated and highly-functional materials, such as those possessing ferroelectric functionality, can be used [12,13]. On the other hand, thermal nanoimprinting is a direct imprinting method used to fabricate nano- and microstructures on polymer films. Therefore, it has the advantage of simplicity in terms of the manufacturing process because of the absence of a coating process [14]. In thermal nanoimprinting, a mold with a nano- or microstructured surface is heated and pressed directly onto a thermoplastic polymer film. The polymer is heated to a temperature above its glass transition temperature (*T_g_*), causing it to flow and fill the nano- or microstructures of the mold. The mold and polymer are then cooled and demolded. Nano- or microstructures can be replicated on polymer films using these simple procedures. This simplicity has increased the popularity of thermal nanoimprinting among researchers as both a promising and convenient method [15].

The mold and polymer require heating and cooling in the thermal nanoimprinting process. The reduction in cycle time is thus limited by the thermal conductivities and specific heat capacities of the mold and polymer. On the other hand, in laser-assisted nanoimprinting, which was developed by Chou et al., nano- or microstructures are replicated by heating only the surfaces of a mold and a substrate using a laser [16]. Xia et al. and Grigalinūnas et al. replicated the nanostructures of a glass mold on a thermoplastic polymer film [17,18]. Grigalinūnas et al. also demonstrated nanoimprint lithography by using an Si mold and a CO_2_ laser [19]. Nagato et al. fabricated nanostructures of diamond-like carbon on a glass mold and replicated them on thermoplastic polymer films in a predefined area by scanning with a laser. They also demonstrated that laser heating reduced the cycle time for thermal imprinting below that associated with conventional thermal nanoimprinting [20].

**Figure 1:**
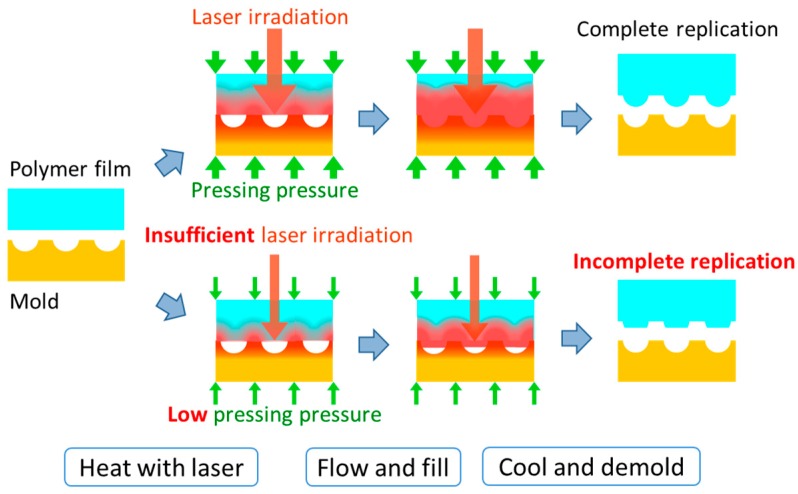
Schematic showing complete and incomplete replication.

However, although it has already been demonstrated that various nanostructures could be replicated on polymer films using laser-assisted thermal imprinting (LATI), this has only been achieved in a few instances. When microstructures are replicated on a polymer film using LATI, the polymer cannot effectively fill the microstructures of the mold, and the microstructures can therefore not be completely replicated. This is because it takes a finite amount of time for the polymer to flow and fill the microstructures of the mold. The flow of the polymer is caused by the applied pressure generated by pressing the mold onto the polymer film and the decrease in viscosity of the polymer is caused by laser heating. Therefore, the pressing pressure and laser-irradiation time both have an important effect on the filling ratio of the polymer (Figure 1).

In this study, microstructures were replicated on polymer films using LATI and the phenomenon of polymer filling was investigated. MLAs with different pattern sizes were used as microstructures and the effects of the pressing pressure, and the pattern size of the MLA, were investigated and the filling ratio was measured by analyzing a microlens replicated using different laser-irradiation times.

## 2. Experimental

### 2.1. MLA Molds

**Figure 2:**
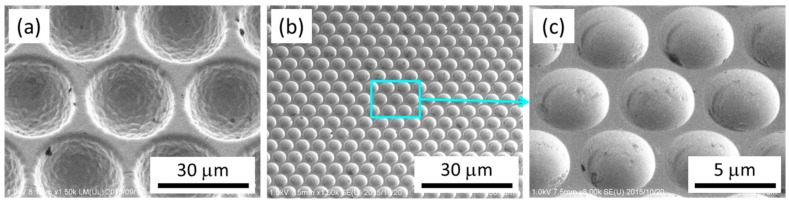
Scanning electron microscope (SEM) images of the microlens arrays (MLAs) in the Ni concave molds all shown as 30°-tilted views. (**a**) 33-μm-pitch MLA, (**b**) 5-μm-pitch MLA, and (**c**) magnified view of 5-μm-pitch MLA.

Figure 2 shows scanning electron microscope (SEM) (semi-inlens field-emission type, SU-8010, Hitachi High-Technologies Corporation, Tokyo, Japan) secondary-electron images of the Ni concave molds with MLAs used in this study. It is possible to fabricate MLAs with various pattern sizes, which are used to improve the light extraction efficiency of OLEDs [21]. One of the MLAs used in this study had a lens diameter of 30 μm, a lens depth of 14 μm, and a pitch of 33 μm, and the other had a spherical lens diameter of 4.2 μm, a lens depth of 2.1 μm, and a pitch of 5 μm. The microlenses were arranged in a regular triangular lattice on both MLAs. The Ni concave molds for the MLAs were constructed using electroforming on convex master molds. Each Ni concave mold had a size of 10 × 10 mm^2^ and a thickness of 200 μm. The convex master molds for the electroforming process were prepared using photolithography and etching on a glass substrate. The mold surfaces were treated with a lubricant for the nanoimprinting mold, which was a hydrophobic coating material (DURASURF, Harves Co. Ltd., Saitama, Japan). A polymethylmethacrylate (PMMA) film was used as a thermoplastic film. The PMMA film had a thickness of 75 μm and *T_g_* of approximately 89 °C (HBA002P, ACRYPLEN, Mitsubishi Chemical Corporation, Tokyo, Japan).

### 2.2. Experimental Setup and Conditions

**Figure 3:**
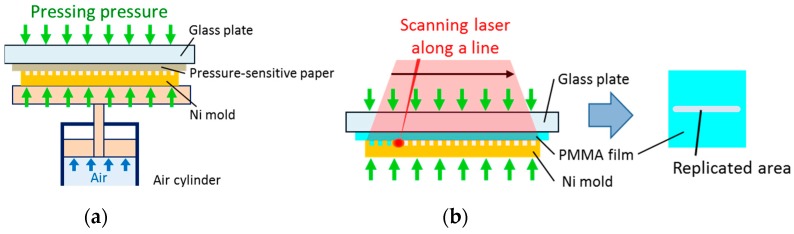
Schematics of (**a**) measurement of the pressing pressure and (**b**) replication by scanning the laser along a line.

Figure 3 shows the experimental setup for the LATI process. A pressure-sensitive paper (Prescale MS R270 10M, of range: 10–50 MPa and accuracy: <±10%, Fujifilm Corporation, Tokyo, Japan) was used to measure the pressing pressure. The pressure was applied using an air cylinder (Misumi, Tokyo, Japan). The pressure-sensitive paper was placed on the Ni concave mold, and the paper and mold were pressed onto a glass plate with a thickness of 15 mm. The pressure applied to the pressure sensitive paper was then measured. In this study, the pressure measured using the pressure-sensitive paper was defined as the pressing pressure. The pressing pressure was set to 10, 20, 30, 40, or 50 MPa by adjusting the air pressure in the cylinder. The above procedure was conducted for each Ni concave mold [11].

After the measurement of the pressing pressure, the pressure-sensitive paper was removed and a PMMA film was placed on the Ni concave mold. The PMMA film and mold were then pressed onto the glass plate with pressing pressures of 10, 20, 30, 40, or 50 MPa. A continuous-wave single-mode fiber laser (SPI Lasers, Southampton, UK) with a wavelength of 1070 nm, 100 W power, and 500 μm diameter was used in this study to irradiate the surface of the Ni concave mold. After the laser was scanned once along a line of length 10 mm, the PMMA film was switched to an unimprinted one and the laser-irradiation time was varied by varying the scanning speed. The scanning speeds were 240, 320, and 500 mm/s for the 33-μm-pitch MLA and 1000, 2000, and 5000 mm/s for the 5-μm-pitch MLA. The laser irradiation-time was defined as the laser-irradiation diameter divided by the scanning speed. The laser-irradiation times were calculated to be 1.0, 1.6, and 2.1 ms for the 33-μm-pitch MLA and 0.10, 0.25, and 0.50 ms for the 5-μm-pitch MLA. Then, for each condition, the MLA replication on the PMMA film was analyzed using a laser microscope (OLS4100, Olympus Corporation, Tokyo, Japan) and the filling ratio of the PMMA was calculated. The height within each cell of area 0.125 × 0.125 μm^2^ was obtained using the laser microscope. The product of the height and area of each cell was then summed for each replicated microlens, and the sum was defined as the volume of the replicated microlens. The volume of the microlens was calculated for each concave master mold. The filling ratio was then defined as the volume of the replicated microlens as a percentage of the volume of the microlens on the concave master mold.

## 3. Results and Discussion

### 3.1. Replication of 33-μm-Pitch MLA

**Figure 4:**
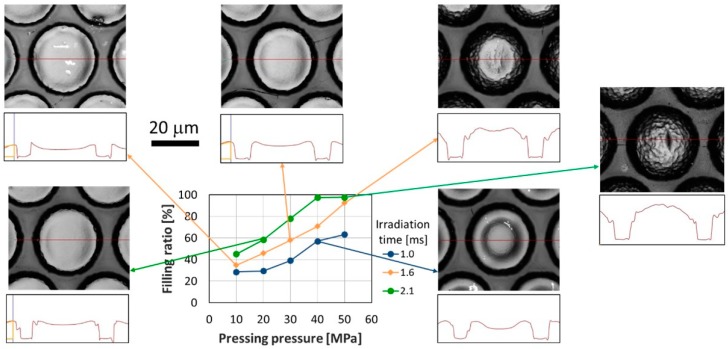
Filling ratio as a function of pressing pressure for each irradiation time in the replication of the 33-μm-pitch MLA.

Figure 4 shows the relationship between the filling ratio and the pressing pressure for each laser-irradiation time in the replication of the 33-μm-pitch MLA. In addition, Figure 4 shows laser microscopic images taken at pressing pressures of 10, 30, and 50 MPa for the laser-irradiation time of 1.6 ms, an image taken at a pressing pressure of 40 MPa and laser-irradiation time of 1.0 ms, and an image taken at a pressing pressure of 20 MPa and laser-irradiation time of 2.1 ms. The filling ratio increased with increasing pressing pressure and laser-irradiation time. This tendency showed that the higher the pressing pressure, the faster the PMMA flowed and filled the mold. When the pressing pressure was increased, the thermal contact resistance between the PMMA film and the Ni mold decreased. The temperature of the PMMA then increased because of the increase in heat input, and the viscosity of the PMMA decreased. This decrease in the viscosity allowed the PMMA to flow more easily. At a pressing pressure of 50 MPa, although the filling ratio was approximately 100% for the laser-irradiation times of 1.6 and 2.1 ms, it was only approximately 60% for the laser-irradiation time of 1.0 ms. This was due to the laser irradiation being insufficient for the laser-irradiation time of 1.0 ms. When the laser irradiation was insufficient, the depth of the PMMA experiencing a temperature exceeding *T_g_* was smaller than the lens depth of the 33-μm-pitch MLA. The filling ratio was almost identical when the pressing pressure and laser-irradiation time were 40 MPa and 1.0 ms, 30 MPa and 1.6 ms, and 20 MPa and 2.1 ms, respectively. In the laser microscopy images obtained under these conditions, the height difference between the highest area and the center of the replicated microlens decreased with increasing laser-irradiation time. This tendency showed that the temperature of the PMMA exceeded *T_g_* not only in the mold contact area but also in the noncontact area when sufficient laser irradiation was provided, causing the PMMA to flow in both the contact area and the noncontact area. On the other hand, when the laser irradiation was insufficient, the temperature of the PMMA only exceeded T_g_ in the contact area and the PMMA flowed only in the contact area. As can be seen in the result associated with an irradiation time of 2.1 ms and pressing pressure of 50 MPa, the filling was almost perfect. The LATI process possibly did not reach 100% filling because of shrinkage due to rapid cooling after the laser irradiation. However, the filling was successful using the various pressing pressures applied in this study.

### 3.2. Replication of 5-μm-Pitch MLA

**Figure 5:**
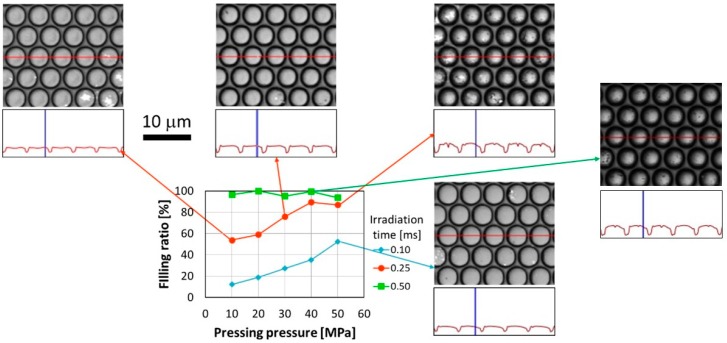
Filling ratio as a function of pressing pressure for each irradiation time in the replication of the 5-µm-pitch MLA.

Figure 5 shows the relationship between the filling ratio and the pressing pressure for each laser-irradiation time in the replication of the 5-μm-pitch MLA. In addition, Figure 5 shows laser microscopic images taken at pressing pressures of 10, 30, and 50 MPa for the laser-irradiation time of 0.25 ms, and an image taken at a pressing pressure of 50 MPa and laser-irradiation time of 0.10 ms. For the laser-irradiation times of 0.10 and 0.25 ms, the filling ratio increased with increasing pressing pressure and laser-irradiation time. For the laser-irradiation times of 0.25 and 0.50 ms, the filling ratio decreased for some pressing pressures. These decreases in the filling ratio may have been due to calculation errors because the PMMA filled the mold almost completely according to the laser microscopy image obtained at a pressing pressure of 50 MPa and laser-irradiation time of 0.25 ms. The filling ratio was approximately 60% at a pressing pressure of 50 MPa and laser-irradiation time of 0.10 ms. This is because the laser irradiation was insufficient for the laser-irradiation time of 0.10 ms. The filling ratio was almost identical when the pressing pressures and laser-irradiation times were 50 MPa and 0.10 ms, and 10 MPa and 0.25 ms, respectively. The laser microscopy images obtained under these conditions showed that the height difference between the highest area and the center of a replicated microlens was almost identical. This indicated that the temperature of the PMMA exceeded *T_g_* not only in the mold contact area, but also in the noncontact area, causing the PMMA to flow from both the contact area and the noncontact area. For an irradiation time of 0.50 ms, the filling ratio was unstable up to approximately 5% throughout the range of pressing pressures. One of the reasons for this is the limitation in accuracy of the laser microscope. A 5% reduction corresponds to a 2% or 3% error in measured length, which equates to 100 or 150 nm error over 5 μm. To investigate this stability, more precise measurements, such as might be attained using an atomic force microscope (AFM), are necessary. The other reason for this 5% disparity is that the heated and low-viscosity area was deeper than shorter irradiation time and the effect of supplying the polymer from underneath and that of escaping the pressure to unirradiated area in planar direction.

### 3.3. Comparison of Surface Shapes during Imprinting

**Figure 6:**
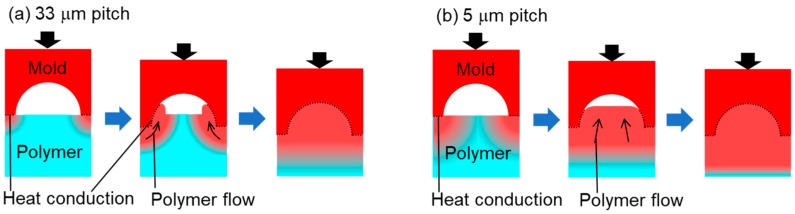
Schematics showing heat conduction and polymer flow during the laser-assisted thermal imprinting (LATI) process applied to (**a**) 33-µm-pitch and (**b**) 5-µm-pitch MLAs.

This section discusses the differences in the shape of the polymer surfaces after undergoing the LATI process. As shown in Section 3.1 and Section 3.2, the leading surface of the polymer in the cavity of the 33- and 5-μm-pitch MLAs showed double and single peaks in the cross-section view after imprinting, respectively. Figure 6 shows the schematic of the heat conduction and polymer flow for the 33- and 5-μm-pitch MLAs. In the larger cavity, the depth of the heated area was smaller than the scale of the cavity and the polymer near the contact surface preferentially decreased in viscosity and a resultant double peak was formed, as shown in Figure 6a. In the smaller cavity, the depth of the heated area was comparable to, or larger than, the scale of the cavity and all the polymer around the contact surface was heated and the resultant polymer flow caused a single peak as shown in Figure 6b. To more comprehensively analyze the polymer flow phenomena seen in Figure 6a,b, experiments should be conducted using MLA with other sizes ranging from between 33 and 5 μm pitch. Applying simulations that incorporate the modelling of viscoelastic body behavior [22,23,24] to the MLA imprinting process would also be useful. Furthermore, other parameters such as laser power, spot diameter, and offset temperature are important and further investigation of these parameters would expand the discussion.

## 4. Conclusions

33- and 5-µm-pitch MLAs were replicated on PMMA films using LATI. The effects of the laser-irradiation time, the pressing pressure, and the pattern size of the MLA were investigated by analyzing the replicated microlens and measuring the filling ratio. In the replication of the 33-µm-pitch MLA, the filling ratio increased with increasing pressing pressure and laser-irradiation time. However, for a laser-irradiation time of 1.0 ms, the PMMA did not fill the mold completely because of insufficient laser-irradiation even though the pressing pressure was increased to 50 MPa, which was the highest pressing pressure applied in this study. Furthermore, the area of the PMMA in which the temperature exceeded *T_g_* and the flow of the PMMA varied with laser-irradiation time. In the replication of the 5-µm-pitch MLA, the filling ratio also increased with increasing pressing pressure and laser-irradiation time. By comparing the surface shapes during filling, it was found that the cross-sectional surface shape possessed a double and single peak in the 33- and 5-μm-pitch pattern, respectively. The depth of heated area of the 33-μm-pitch pattern was smaller than the pattern size, whereas that of the 5-μm-pitch pattern was comparable to or larger than the pattern size. Future experiments will need to be conducted with patterns of other sizes along with simulation modelling of heat conduction and polymer flow to expand on the current discussion. MLA performance characteristics, such as light-extraction efficiency, will also be verified as part of future work.
